
JOURNAL INFORMATION
==============================
NLM Title Abbreviation: Thorac Cancer
Journal ID: Thorac Cancer
NLM Journal ID: 101531441
Journal ID: TCA

Thoracic Cancer

ISSN: 1759-7706
EISSN: 1759-7714
Publisher: Wiley

ARTICLE INFORMATION
==============================
PMCID: PMC11474528
DOI: 10.1111/1759-7714.14514
Article ID: TCA14514
Article version: 1
Subjects: Issue Information, Issue Information

Issue Information

Electronic publication date: 2023 Oct 1
Publication date: 2023 Oct
Volume: 14
Issue: 28
First page: 2811
Last page: 2812
Copyright: © 2023 The Authors. Thoracic Cancer published by China Lung Oncology Group and John Wiley & Sons Australia, Ltd.
License: This is an open access article under the terms of the http://creativecommons.org/licenses/by/4.0/ License, which permits use, distribution and reproduction in any medium, provided the original work is properly cited.
License URL: https://creativecommons.org/licenses/by/4.0/

Figure count: 0
Table count: 0
Page count: 5

source-schema-version-number: 2.0
cover-date: October 2023
details-of-publishers-convertor: Converter:WILEY_ML3GV2_TO_JATSPMC version:6.4.9 mode:remove_FC converted:15.10.2024

==============================

Volume 14, Number 28

October 2023

### Thoracic Cancer

Open Access

Prevention, treatment and research of thoracic cancer in Asia Pacific www.ThoracicCancer.net

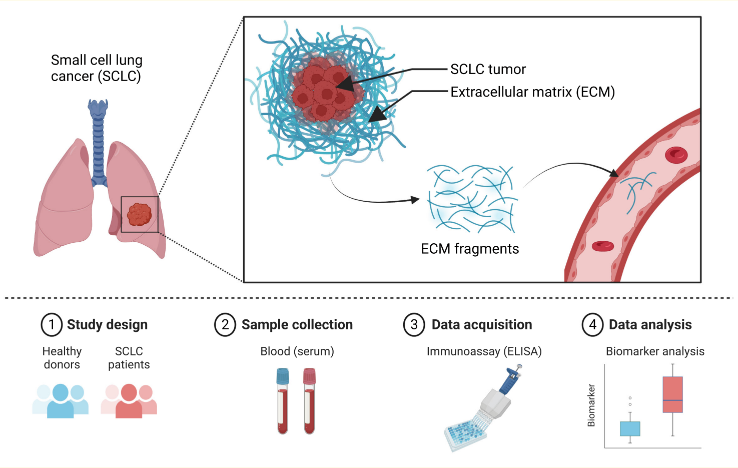

- An optimal dose-fractionation for stereotactic body radiotherapy in peripherally, centrally and ultracentrally located early-stage non-small lung cancer
- Preliminary investigation of elevated collagen and blood-clotting markers as potential noninvasive biomarkers for small cell lung cancer
- Real-world treatment patterns and clinical outcomes after introduction of immune checkpoint inhibitors: Results from a retrospective chart review of patients with advanced/metastatic non-small cell lung cancer in the EU5
- Role of prognostic nutritional index in postoperative radiotherapy for non-small cell lung cancer

WILEY

The official journal of the Chinese Society of Lung Cancer and the International Chinese Society of Thoracic Surgery. Endorsed by the Korean Association for the Study of Lung Cancer and the Hong Kong Cancer Therapy Society

### Thoracic Cancer

Open Access

**Editors-in-Chief**
Qinghua Zhou, *Tianjin, China*
Yan Sun, *Beijing, China*

**Associate Editors**
Shujun Cheng, *Beijing, China*
Young-Chul Kim, *Chonnam, Korea*
Tony Mok, *Hong Kong, China*
Youlin Qiao, *Beijing, China*
Jun Wang, *Beijing, China*

**Editorial Board Members**
Charles Butts, *Edmonton, Canada*
Jun Chen, *Tianjin, China*
Ke-Neng Chen, *Beijing, China*
Liang-An Chen, *Beijing, China*
Wan-Qing Chen, *Beijing, China*
Moon-June Cho, *Daejeon, Korea*
William Chi-Shing Cho, *Hong Kong, China*
Sebastian Dango, *Göttingen, Germany*
Zi-Ding Feng, *Houston, USA*
David Fielding, *Brisbane, Australia*
Jian-Hua Fu, *Guangzhou, China*
Xiao-Long Fu, *Shanghai, China*
Ignasi Garcia-Olivé, *Barcelona, Spain*
Shou-Wei Han, *Louisville, USA*
Sam Hanash, *Houston, USA*
Bao-Hui Han, *Shanghai, China*
Hao Long, *Guangzhou, China*
Jianxing He, *Guangzhou, China*
Jie He, *Beijing, China*
Kwang Ho In, *Seoul, Korea*
Michael Kuan-Yew Hsin, *Hong Kong, China*
Yujin Kudo, *Tokyo, Japan*
Guohong Hu, *Shanghai, China*
Wenlin Huang, *Guangzhou, China*
Hong-Bin Ji, *Shanghai, China*
Stephen Lam, *Vancouver, Canada*
Wan L. Lam, *Vancouver, Canada*
Choon-Taek Lee, *Seoul, Korea*
Weimin Li, *Chengdu, China*
Zhong-Xing Liao, *Houston, USA*
Dong-Xin Lin, *Beijing, China*
Yong Lin, *Albuquerque, USA*
Hui-Ping Liu, *Linko, Taiwan*
Lunxu Liu, *Chengdu, China*
Jiade Lu, *Singapore*
Feng Luo, *Chengdu, China*
Yoshimasa Maniwa, *Kobe, Japan*
Andrew Medford, *Bristol, UK*
Takahiro Nakajima, *Chiba, Japan*
C S Pramesh, *Mumbai, India*
Ghee Chee Phua, *Singapore*
Gaetano Rocco, *Naples, Italy*
Jesse Roman, *Louisville, USA*
Yuan-Kai Shi, *Beijing, China*
Shi-Yong Sun, *Atlanta, USA*
Sook Whan Sung, *Seoul, Korea*
Koji Takeda, *Osaka, Japan*
Paul Van Schil, *Antwerp, Belgium*
Jie Wang, *Beijing, China*
Ko Pen Wang, *Baltimore, USA*
Lu-Hua Wang, *Beijing, China*
Benny Weksler, *Memphis, USA*
Yi-Long Wu, *Guangzhou, China*
Yu-Chung Wu, *Taipei, Taiwan*
Yaguang Xi, *New Orleans, USA*
Xue-Yuan Xiao, *Beijing, China*
Yan Xu, *Beijing, China*
Gary Yang, *Loma Linda, USA*
Tokujiro Yano, *Fukuoka, Japan*
Kazuhiro Yasufuku, *Toronto, Canada*
Jin-Ming Yu, *Jinan, China*
Hongquan Zhang, *Beijing, China*
Hong-Tao Zhang, *Suzhou, China*
Jian Zhang, *Ann Arbor, USA*
Li Zhang, *Guangzhou, China*
Xiu-Yi Zhi, *Beijing, China*
Cai-Cun Zhou, *Shanghai, China*
Wei Zhou, *Atlanta, USA*

**Aims and Scope:** *Thoracic Cancer* aims to facilitate international collaboration and exchange of comprehensive and cutting-edge information on basic, translational, and applied clinical research in lung cancer, esophageal cancer, mediastinal cancer and other thoracic malignancies. Prevention, treatment and research relevant to Asia Pacific are a focus, but submissions from all regions are welcomed. The editors encourage contributions relevant to prevention, general thoracic surgery, medical oncology, radiology, radiation medicine, pathology and basic cancer research as well as epidemiological and translational studies in thoracic cancer. *Thoracic Cancer* is the official publication of the Chinese Society of Lung Cancer, International Chinese Society of Thoracic Surgery, and is endorsed by the Korean Association for the Study of Lung Cancer and the Hong Kong Cancer Therapy Society.

**Editorial Correspondence:**
Dr. Qian Liu, Managing Editor, *Thoracic Cancer*, Room 621, Scientific Research Building, National Cancer Center/National Clinical Research Center for Cancer/Cancer Hospital, Chinese Academy of Medical Sciences and Peking Union Medical College, Beijing, 100021, China

Email: TCA50@wiley.com; liuqian2022@cicams.ac.cn; liuqian@lungca.org

Tel: 86 10 8778 8831

Fax: 86 10 8778 8831

**Disclaimer:** The Publisher, China Lung Oncology Group and Editors cannot be held responsible for errors or any consequences arising from the use of information contained in this journal; the views and opinions expressed do not necessarily reflect those of the Publisher, China Lung Oncology Group or Editors, neither does the publication of advertisements constitute any endorsement by the Publisher, China Lung Oncology Group or Editors of the products advertised.

**Copyright and Copying (in any format)**
Copyright © 2023 China Lung Oncology Group and John Wiley & Sons Australia, Ltd. All rights reserved. No part of this publication may be reproduced, stored or transmitted in any form or by any means without the prior permission in writing from the copyright holder. Authorization to copy items for internal and personal use is granted by the copyright holder for libraries and other users registered with their local Reproduction Rights Organisation (RRO), e.g. Copyright Clearance Center (CCC), 222 Rosewood Drive, Danvers, MA 01923, USA (www.copyright.com), provided the appropriate fee is paid directly to the RRO. This consent does not extend to other kinds of copying such as copying for general distribution, for advertising or promotional purposes, for republication, for creating new collective works or for resale. Permissions for such reuse can be obtained using the RightsLink “Request Permissions” link on Wiley Online Library. Special requests should be addressed to: permissions@wiley.com

**Copyright © 2023 China Lung Oncology Group and John Wiley & Sons Australia, Ltd**

**Cover Image:** Photo courtesy of Jeppe Thorlacius-Ussing et al. Permission for reproduction of the cover image is gratefully acknowledged.

**For submission instructions and all other information visit: www.thoraciccancer.net**

ISSN 1759-7706 (Print)
ISSN 1759-7714 (Online)
